# Signaling pathways of dental implants’ osseointegration: a narrative review on two of the most relevant; NF-κB and Wnt pathways

**DOI:** 10.1038/s41405-024-00211-w

**Published:** 2024-04-05

**Authors:** Samar Mohamed Emam, Nermine Moussa

**Affiliations:** 1 Department of Prosthodontics, Faculty of Dentistry, Alexandria University, Alexandria, Egypt; 2 Present Address: Department of Biotechnology, Institute of Graduate Studies and Research, Alexandria University, Alexandria, Egypt

**Keywords:** Peri-implantitis, Oral pathology, Removable prosthodontics

## Abstract

**Introduction:**

Cell signaling pathways are the biological reactions that control cell functions and fate. They also directly affect the body reactions to implanted biomaterials. It is well-known that dental implants success depends on a successful integration with the alveolar bone: “osseointegration” which events comprise early and later responses to the implanted biomaterials. The early events are mainly immune-inflammatory responses to the implant considered by its microenvironment as a foreign body. Later reactions are osteogenic aiming to regulate bone formation and remodeling. All these events are controlled by the cell signaling pathways in an incredible harmonious coordination.

**Aim:**

The number of pathways having a role in osseointegration is so big to be reviewed in a single article. So the aim of this review was to study only two of the most relevant ones: the inflammatory Nuclear Factor Kappa B (NF-κB) pathway regulating the early osseointegration events and the osteogenic Wnt pathway regulating later events.

**Methods:**

We conducted a literature review using key databases to provide an overview about the NF-κB and Wnt cell signaling pathways and their mutual relationship with dental implants. A simplified narrative approach was conducted to explain these cell signaling pathways, their mode of activation and how they are related to the cellular events of osseointegration.

**Results and conclusion:**

NF-κB and Wnt cell signaling pathways are important cross-talking pathways that are affected by the implant’s material and surface characteristics. The presence of the implant itself in the bone alters the intracellular events of both pathways in the adjacent implant’s cellular microenvironment. Both pathways have a great role in the success or failure of osseointegration. Such knowledge can offer a new hope to treat failed implants and enhance osseointegration in difficult cases. This is consistent with advances in Omics technologies that can change the paradigm of dental implant therapy.

## Introduction

### Cell signaling pathways

Cell signaling pathways are the key biological mechanisms that explain how cells respond to extracellular signals in order to regulate intracellular gene expression [1, 2]. They comprise cellular biochemical reactions that transfer information from a receptor to a target in the nucleus or mitochondria [3]. Signal transduction pathways affect distinct classes of molecules that collectively determine the response of a given cell to its afferent autocrine, endocrine or paracrine signals [4, 5]. This is achieved by the binding of a specific ligand to the corresponding membrane-bound receptor, triggering a cascade of intracellular signaling activities through multiple kinases, affecting how transcription factors regulate downstream gene expression. This results in a change in gene expression and/or protein activation levels leading to phenotype changes [1–3].

Pathways were thought to be a linear series of reactions. Recent studies discovered that they are a complex series of non-linear reactions. Many of them are enzyme catalyzed protein activations, often arranged in a cascade form where the activated protein on one level is the enzyme activating the reaction on the next level. The interaction between different pathways is known in life sciences as “cross-talk”. Bioinformatics modeling studies on signaling pathways and their cross-talk defined five different types of cross-talk [3]. A novel research -the Signaling Pathways Project (SPP)- found that mammalian signal transduction pathways comprise four major categories of pathway module, all collaborate to initiate, propagate and give effect to the signals through gene expression [4, 6].

The harmonious coordinated activity between signaling pathways promotes numerous cellular responses forming the basis of important biological processes, such as development, tissue repair and immunity. Any interruption within these extra and/or intra-cellular communication chains can cause diseases such as developmental disorders, inflammatory diseases and cancers. Therefore, understanding the activity and the cross-talk between signaling pathways can help to design novel disease treatments and tissue regeneration strategies [1, 7, 8]. This can also help to understand and overcome the problems of different biomaterials implanted in the body for therapeutic and/or diagnostic purposes, inducing a highly complicated series of immune-modulating interactions with the host immune system and its signaling pathways [9, 10]. This can be applied to all body implants, including dental implants.

### Osseointegration of dental implants

Dental implants are widely used in dental medicine as a reconstructive treatment modality to restore full or partial loss of teeth. The success of oral implantation depends on successful overlapping phases of tissue healing and regeneration at the implant-bone interface, to finally get a dental implant that is “osseointegrated” with the alveolar bone. This happens through a complex cascade of biological events providing a direct structural and functional connection between the surface of a load-bearing implant and living bone [11, 12]. Titanium has been the material of choice for dental implants. Zirconia and polyether-ether-ketone (PEEK) has become recent alternatives [11, 13]. However, this review will focus on the cellular events and signaling pathways related to the osseointegration of Titanium dental implants.

Titanium implants have been perceived as chemically and biologically inert materials which should be non-reacting with their biological environment. Therefore, when placed in living tissues such as bone and gingiva, they would be integrated with them through a pure wound healing process and osseointegration process would be a pure wound healing phenomenon. In fact, clinicians and material scientists realized that inertness was not synonymous with biocompatibility. Recent studies revealed that any material that penetrates the body tissues activates the immune system, therefore biomaterials are immunomodulatory rather than inert [10, 14, 15]. Hence, osseointegration must be considered as an immune-modulated inflammatory process, where the immune system is locally either up or down-regulated, largely influencing the whole healing process [10].

Osseointegration starts by being an actual foreign body reaction (FBR) to biomaterials. Its ultimate goal is to shield off the foreign implant material through complex healing process. Cellular events start by immune-modulated inflammatory response followed by bone formation and remodeling [10, 16]. Peri-implant immune cells play an important role in the local microenvironment since a proper osteoimmunomodulation is essential to reach a favorable osseointegration. Angiogenesis is another important component of a successful osseointegration. Peri-implant osteogenesis is essential for the stability and function of dental implants. It is regulated by the dynamic balance between osteoblasts, osteoclasts and osteocytes [17–19]. Finally, a foreign body reaction balance or equilibrium (FBE) is achieved with successful implant bone integration [10, 16].

But this is not the end, in fact, loaded titanium implants are rarely totally covered by mineralized bone. Instead, the cells characteristic of a typical FBR -such as macrophages and foreign body giant cells- are present at the implant-bone interface. Different stimuli affect these cells, along with osteocytes, keeping them biologically active, resulting in bone degradation and/or deposition. Therefore, osseointegration is considered a dynamic event rather than a static one [10, 20, 21]. Understanding the osteoimmunology of the peri-implant environment is essential: initial inflammatory innate immune response is critical for bone formation then the adaptive immune system -activated by continuous release of Titanium ions in the peri-implant environment- also seems to be involved in the peri-implant tissue healing [22].

We should note that the presence of an implant appears to be a significant factor modulating gene networks in the peri-implant tissue. Complex molecular pathways with variations in gene expression for different reasons play important roles in osseointegration [23]. A whole genome transcriptional analysis revealed that gene expression varies along the course of osseointegration. Early stages involve expression of immuno-inflammatory response associated genes. These are then replaced by genes associated with the biological processes of osteogenesis, angiogenesis and neurogenesis. Therefore, early immuno-inflammatory changes appear to be regulated via the I-κB kinase/NF-κB cascade, whereas the later osteogenesis-related mechanisms are regulated by TGF-β/BMP, Notch and Wnt signaling [24].

The aim of this review is to study signaling pathways that are most relevant to osseointegration. A narrative review approach will be taken to review our current knowledge on the pro-inflammatory NF-κB signaling pathway and the osteogenesis-related Wnt signaling pathway in relation to osseointegration of dental implants and how this can be used to improve the clinical outcomes of dental implants’ therapy.

## Nuclear Factor Kappa B pathway: a master inflammatory regulator of osseointegration

### What is NF-κB

#### History, nomenclature and functions

Nuclear factor kappa B is a master regulatory protein of many evolutionarily conserved cellular biochemical reactions. It was discovered in 1986 as a nuclear factor that binds to the enhancer element of kappa light-chain of the immunoglobulin of activated B cells, hence the abbreviation NF-κB. Later, it was found that NF-κB proteins are a group of transcription factors expressed in nearly all cell types and regulate many target genes with a whole variety of functions. They organize cellular resistance against pathogenic signals through the complex network of NF-κB signaling pathway. Therefore, they regulate the innate immunity, inflammatory response, oncogenesis, nervous system function and metabolic disorders [25–27]. NF-kB functions extend to control the embryonic development and physiology of bone, skin, and central nervous system [28, 29].

#### Structure and regulation

The NF-κB family of transcription factors is formed by dimerization of two of its five structurally related members: NF-κB1 (p50), NF-κB2 (p52), RelA (p65), RelB and c-Rel [30, 31]. In the absence of stimuli, most NF-κB dimers in the cytoplasm are bound to inhibitory molecules of the IκB family of proteins (inhibitors of NF-κB). IκBs are characterized by ankyrin repeats that bind to DNA-binding domains of NF-κB proteins making them transcriptionally inactive then sequestered in the cytoplasm. The most important IκB family member is the IκBα protein [27, 30]. In fact,the master regulator of NF-κB signaling pathway is the high molecular weight protein IκB kinase complex (IKK) that is composed of two catalytic subunits, IKKα (IKK1) and IKKβ (IKK2), and a regulatory IKKγ subunit named NF-κB essential modulator (NEMO) [32].

### Activation of NF-κB pathway and its target genes

#### Causes of activation

NF-κB is a pro-inflammatory signaling pathway based on the activation of NF-κB proteins by pro-inflammatory cytokines such as interleukin 1 (IL-1) and tumor necrosis factor α (TNFα) that are rapidly released due to infection and activate toll-like microbial pattern recognition receptors (TLRs). In the absence of infection, TLRs are recognized by endogenous ligands that may trigger inflammation associated with tissue injury and certain diseases [33–35].

#### Modes of NF-κB activation

There are two separate pathways for NF-κB activation: The “canonical” or classical pathway and the “noncanonical” or alternative pathway that vary in their mode of activation. Both pathways require different triggers and stimuli to be activated. Furthermore, the genetic analysis of their master regulator -the IKK complex- has identified that both pathways differ in their requirement for IKK subunits.

The canonical pathway is triggered by microibial lipopolysaccharides and proinflammatory cytokines such as TNFα and IL-1 activating corresponding receptors. Hence, an excitatory signaling is mediated leading to activation of IKKβ unit in the IKK complex. The IKKβ subunit phosphorylates IκBα in a reaction that requires the IKKγ (NEMO) regulatory subunit. This phosphorylation is essential for the polyubiquitination of IκBα resulting in its proteosomal degradation. Then a rapid nuclear translocation occurs of canonical NF-κB members predominantly the p50/RelA and p50/c-Rel dimers leading to their activation as transcription factors with target genes expression [27, 30, 32, 33].

The alternative pathway selectively responds to a specific group of stimuli, including endogenous ligands of a subset of TNF Receptors superfamily members, other than the TNFα. These include the CD40 ligand, B cell activating factor receptor (BAFF-R) ligand and receptor activator of NF-κB ligand (RANKL) activating corresponding receptors leading to activation of IKKα in the IKK complex by the NF-κB-inducing kinase (NIK) [27, 30, 33]. The IKKα phosphorylates p100, the precursor protein of NF-κB2 leading to polyubiquitination of p100 and its proteasomal processing to mature p52. The noncanonical NF-κB activation does not involve IκBα degradation. Processing and maturation of P52 is followed by nuclear translocation of noncanonical NF-κB complex p52/RelB and transcriptional activation of target genes [27, 36–38].

#### NF-κB target genes

NF-kB transcription factors control a large number of target genes that are central components of the immune response. They regulate the transcription of the genes encoding cytokines, chemokines and cell adhesion molecules. Factors of the complement cascade, immune receptor subunits and major histocompatibility complex (MHC) molecules are also NF-kB dependent. Moreover, the regulators of NF-kB such as the IkBα and other molecules are themselves NF-kB dependent forming an auto-regulatory feedback loop [28, 33]. NF-kB dependent target genes also include genes regulating cell proliferation and apoptosis. Hence, a lot of important cellular processes are regulated through NF-kB-dependent transcription and any dysregulation of this pathway results in many diseases such as autoimmune diseases and cancer [28, 29].

### NF-κB pathway and osseointegration of dental implants

Osseointegration is an immune-modulated complex healing process that represents a foreign body reaction to biomaterials. It is a multifactorial process that depends on the interaction between the implant material and surface treatment and the cells present in the implant’s microenvironment aiming to reach a foreign body equilibrium status [10]. In this section of the review, we will discuss:

-How the NF-κB signaling is affected by the implant’s structural variations, insertion torque and loading.

-The NF-κB-dependent role of macrophages and osteoclasts in osseointegration.

-How the NF-κB signaling varies and alters osseointegration with variations in the patient’s metabolism and genetic factors.

#### Effect of implant material, topography and surface modification on NF-κB

As soon as a dental implant is inserted, a specific protein adsorption pattern is formed onto Titanium surface modulating the immune response with activation of NF-κB, the master inflammatory transcription factor. The major signaling pathway positively regulated at the 4^th^ day of dental implant insertion was the I-κB kinase/NF-κB cascade. Then, bone and immune cells interact till reaching a FBE-mediated osseointegration [22, 24]. Such immune response to dental implants can be manipulated through beneficial “osteoimmunomodulation”. This can be achieved through various surface modifications of dental implants [22, 39].

The implant surface material, topography and composition have a great impact on the behavior of the adjacent immune and bone cells [40]. Titanium as a biomaterial has been recently considered immunomodulatory rather than inert. This may be related to surgical trauma and external stresses activating corrosion behavior leading to Ti ions released over time around the implant. Ti ions form an inflammatory microenvironment through activation of the NF-κB pathway in the adjacent macrophages [10, 41]. Moreover, surface roughness such as sandblasted large-grit acid etching (SLA) tends to increase the proinflammatory response that can be reduced by chemically modified SLActive surfaces [40]. Earlier surface modification studies including fluorohydroxyapatite (FHA) coated titanium alloy implants detected slight improving in the cellular activity promoting osteogenesis on FHA-coated materials in comparison with their control [42, 43]. Recently, the nanoscale and chemical modification of implant surfaces with the resultant effect on osteogenic capability has gained more attention [44].

One example of implants chemical surface modification is the creation of a thick, porous oxide layer on the titanium implant by means of anodic oxidation. This modification resulted in a higher expression of RANK and RANKL. Therefore, a higher early osteoclastic differentiation was induced, with intensive bone remodeling accelerating the maturation of the bone-implant interface [39]. An example of nanoscale surface modification was coating Titanium implants with chitosan-gold nanoparticles conjugated with c-myb, a transcription factor that supports bone formation. Plasmid DNA/c-myb conjugated with chitosan-gold nanoparticles (Ch-GNPs/c-myb) promoted osteogenesis and inhibited osteoclastogenesis through downregulation of RANKL and reducing the nuclear translocation of NF-κB [45].

#### NF-κB-induced bone remodeling around dental implants before and after loading

As soon as an implant is placed, it is passively stabilized in the bone through frictional primary bone contacts creating a mechanical primary stability that is essential for successful osseointegration. An adequate implant insertion torque (IT) -25 to 45 Ncm- ensures a microenvironment that prevents fibrous encapsulation. In the same time, a high IT causes an increased pressure at the bone implant interface creating microfractures and bone necrosis by direct harming effect on the osteocytes [40, 46].

Osteocytes are stellate-shaped cells located within lacunae and surrounded by mineralized bone matrix. They are terminally differentiated osteoblasts that regulate bone remodeling by modulating osteoclast and osteoblast activities [47]. When the insertion torque is increased above optimal level, bone microcrofractures result in osteocytes injury and apoptosis. Dying osteocytes release signals raising RANKL levels to activate osteoclasts. High RANKL and low OPG levels have been reported 150–200 µm away from microcracks [48, 49]. This explains the marginal bone loss when the implant is placed under high IT (50 Ncm or more) in spite of excellent primary stability [40].

The mechanosensitive osteocytes have a notable role in bone remodeling after implant loading and restoration delivery. It is well known that the lack of mechanical loading creates a state of bone disuse activating bone lining cells expressing RANKL and activate osteoclatogenesis and bone resorption. Mechanical loading is sensed by the osteocytes that send molecular signals coupling bone formation and resorption. An adequate functional loading increases bone formation and inhibits resorption while excessive loading leads to bone microdamage with higher osteocytes apoptosis, increased RANKL and bone resorption [40, 48, 50].

#### The NFκB-related role of macrophages in osseointegration

Dental implants placement triggers a specific immunoinflammatory response that modulates early phases of wound healing. In fact, the inflammatory response is differentially regulated during osseointegration since the presence of titanium itself can influence the gene expression of inflammatory cells [44]. It is well known that macrophages play an important role in the bone homeostasis and bone-biomaterial integration around dental implants. They are “plastic” cells that can polarize from classical pro-inflammatory M1 macrophages towards anti-inflammatory tissue regenerative M2 macrophages [40, 51].

NF-κB is a key transcription factor of M1 macrophages. Its target genes include a large number of inflammatory genes such as those encoding TNF-α, IL-1β, IL-6, IL-12p40 and cyclooxygenase-2 [30, 52]. Meanwhile, the anti- inflammatory pro-regenerative M2 macrophages produce Wnt ostegenic ligands [22]. The balance between M1/M2 macrophages is important in wound healing, tissue regeneration and osseointegration. The macrophages polarization between both phenotypes depends on the implant microenvironment, foreign body reaction and the invading microorganisms [22, 40].

Osteal macrophages (OsteoMacs) are a specific subset of macrophages present in bone tissues. Recently, it has been hypothesized that osteomacs play an essential role in implant osseointegration [40, 53]. Studies accompanied with removal of osteomacs were associated with reduction in bone remodeling and repair that is also a major function of osteoclasts [40, 54]. However, although derived from common myeloid progenitor cell precursors and can be stimulated by the same cytokines, osteal macrophages and osteoclasts are not synonymous. They can be differentiated through the mature macrophage marker CD169 [55]. Osteomacs and osteoclasts are similar in being RANKL-induced differentiated [22].

#### Role of NF-κB in osteoclastogenesis

Osteoclasts are bone resorption cells differentiated from monocytes and macrophages. Their main differentiation factor is the Receptor activator of NF-κB ligand (RANKL) [56]. The role of IKK1, IKK2 and NEMO in osteoclasogenesis was studied on animal models. It was found that NF-κB activation, in response to RANKL or TNF activates the osteoclast differentiation factors c-Fos and NFATc1 that are important for the final osteoclast differentiation and activity. NF-κB signaling mediates oral infections and periodontitis since bacterial lipopolysaccharides activate NF-κB, osteoclastogenesis, and osteolysis [57]. History of periodontal disease is closely related to the prevalence of peri-implantitis [40].

As soon as a dental implant is placed, cellular events start by hemostasis (exudative phase), followed by inflammatory phase, proliferative phase and finally the remodeling phase [46]. This physiological bone remodeling around dental implants is actually a foreign body reaction led by RANKL promoting macrophages activation into osteoclasts. The bone lining cells (BLCs) exert also an important function during bone resorption through their ability to express RANKL promoting osteoclastogenesis. BLCs modulate bone remodeling by digesting the protrusive non-mineralized collagen fibers mediated by matrix metalloproteinases (MMPs), hence cleaning the bone surface in order to facilitate osteoclast attachment that is followed by the resorption process [40, 58].

#### Effect of metabolic and genetic factors of the implant’s host on NF-κB

The implant’s host characteristics modulate the interaction between the implant and its microenvironment. Therefore, the cascade of NF-κB-dependent immune responses triggered after implant placement vary according to the host metabolic and genetic factors. The metabolic factors are systemic bone remodeling factors affecting local peri-implant bone metabolism while the genetic factors are inherited personal DNA variations [10, 40]. Body fat and vitamin D levels are the main metabolic factors affecting osseointegration through the NF-κB pathway [40].

##### Metabolic factors

The relationship between bone and body fat is complex and not fully understood. However, it was found that the bone marrow fat is inversely related to the bone mass. This is due to two main points: the first is the increased number of adipocytes in the bone marrow secreting more saturated fatty acids impairing osteoblast vitality by inducing autophagy and apoptosis. The second point is that the bone marrow adipocytes release more pro-inflammatory and osteoclastogenic cytokines (e.g., TNFα and IL-6) and adipokines with more expressed RANKL and more activation of the NF-κB pathway [40, 59].

The role of vitamin D in the prevention and treatment of osteoporosis is well established but research investigating its effects during dental implant osseointegration remains limited. Implant failure has been related to the insufficiency of vitamin D prevalence in various patient populations [40]. An adequate serum vitamin D level is essential for the success of Ti implants. This has been explained through studying the circadian rhythm pathway and the expression of Neuronal PAS domain-containing protein 2. *NPAS2* gene was the most significantly affected gene by vitamin D deficiency in peri-implant tissue in a rat model study [40, 60]. Regarding its role in inflammation, it was found that vitamin D reduces inflammation by inhibiting NFκB activity since vitamin D receptor (VDR) interacts with IKKβ to block NF-κB pathway activation. Also, vitamin D inhibits the TNFα-induced p65 nuclear translocation attenuating the NF-κB activity in a VDR-dependent manner [61, 62]. This can explain the enhanced implant healing and reduced failure with vitamin D supplementation. Further studies are needed.

##### Genetic factors

It was noted that osseointegration failure may occur in a “cluster” form, i.e. failure of multiple implants in the same patient. This clustering nature of implant failure can be attributed to a genetic predisposition affecting the immune response. Single nucleotide polymorphisms (SNPs) represent the most common genetic variations. A non-synonymous SNP in a protein coding region results in an altered phenotype with a different structural and functional property [63, 64].

Polymorphisms in the *RANK* and *RANKL* genes were studied to find their association with implant failure. The CC genotype of rs35211496 SNP in *RANK* gene and CT genotype of rs9533156 SNP in *RANKL* gene were significantly associated with peri-implantitis and suggested as genetic risk factors [63, 65, 66]. Other genetic association studies can be indirectly relevant to the NF-κB pathway. For example, the Osteoprotegerin (OPG) is a cytokine receptor that, along with RANKL, antagonizes the RANK-mediated activation of NF-Kβ and reduces bone resorption. The CC genotype of rs2073618 SNP in *OPG* gene and the G-C haplotype frequency of rs2073618-rs2073617 in the same gene were significantly associated with peri-implantitis in a sample Chinese Han population [67, 68].

**Fig. 1:**
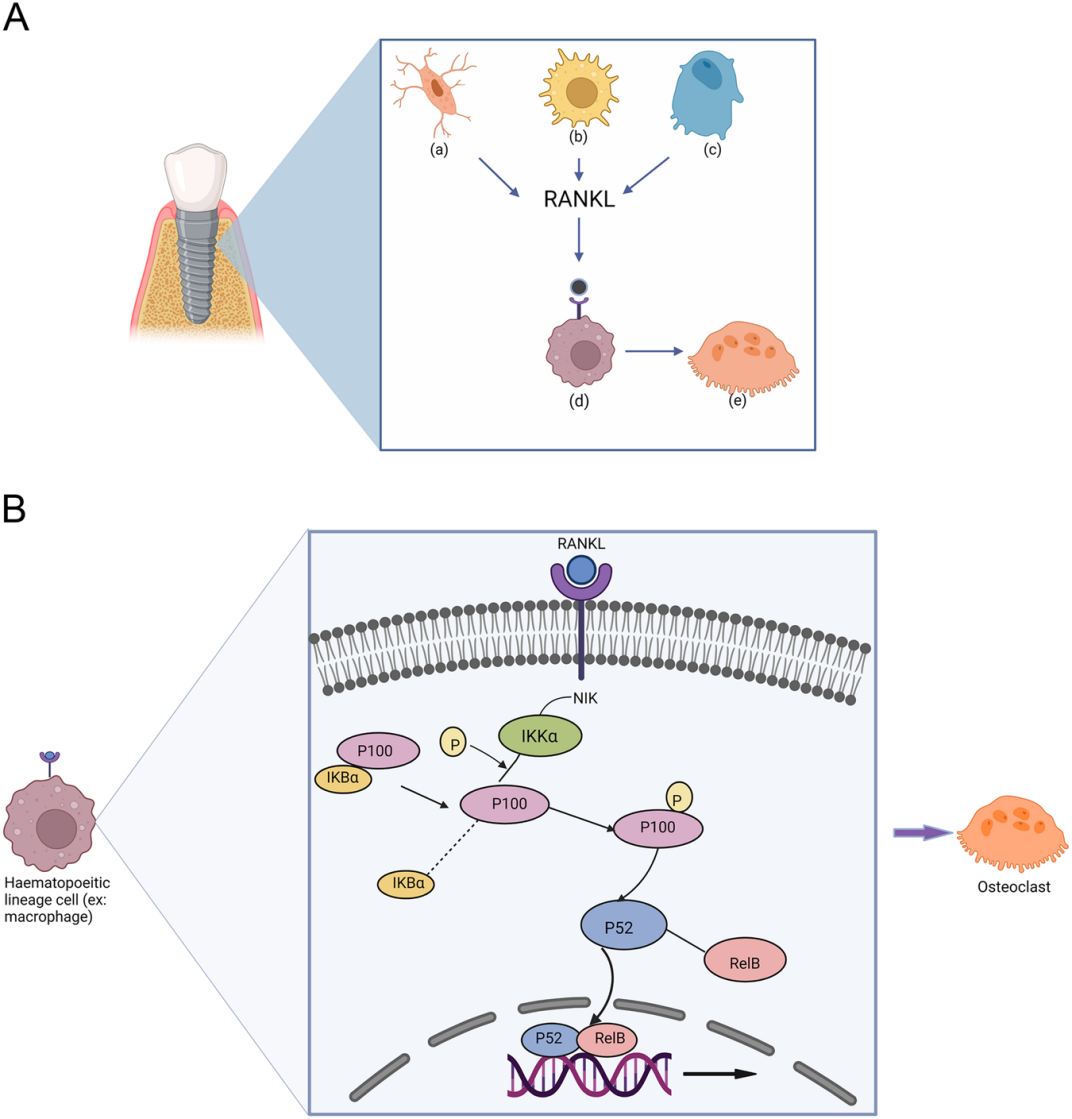
Non-canonical activation of NF-κB pathway and RANKL-based osteoclastogenesis around dental implants. **A**, **B** Summarizes the role of RANKL-based activation of NF-κB in osteoclatogenesis. **A** Diagrammatic representation of a dental implant surrounded by alveolar bone during early inflammatory osseointegration events. At the implant bone interface, (a) osteocytes, (b) activated T cells and (c) osteoblasts secrete receptor activator of nuclear factor kappa-B ligand (RANKL) responsible for osteoclasts differentiation from haematopoietic lineage cells such as (d) macrophages. RANKL attaches to its receptor (RANK) on the cell membrane and activates the alternative NF-κB pathway inside the cell leading to its maturation into an (e) osteoclast. Created with BioRender.com. **B** Activation of receptor activator of NF-κB (RANK) at the cell membrane leads to the activation of α subunit of I*κ*B kinase complex (IKKα) by the NF-κB-inducing kinase (NIK). The IKKα phosphorylates p100, the precursor protein of NF-κB2 (p52) leading to polyubiquitination of p100 and its proteasomal processing to mature p52. The noncanonical NF-κB activation does not involve degradation of the inhibitory molecule IκBα that was attached to p100 before its activation. Processing and maturation of P52 is followed by nuclear translocation of noncanonical NF-κB complex p52/RelB and transcriptional activation of target genes. Created with BioRender.com.

Figure 1A and B summarizes the role of RANKL-based activation of NF-κB in osteoclatogenesis.

## Wnt pathway: a master regulator of osteogenesis and implant’s osseointegration

### What is Wnt

#### Nomenclature and function

Wnt signaling pathway is an evolutionarily conserved pathway that was studied in Drosophila before mammals. Hence its name is resulting from the fusion of the name of Drosophila segment polarity gene *wingless* and the name of its vertebrate homolog, *integrated (int-1)*.

Wnt pathway regulates embryonic developmental processes such as cell fate determination, cell migration, polarity, neural patterning and organogenesis. It is also a key regulator of multiple postnatal health and disease conditions. Variations in Wnt proteins can result in defects in embryonic development. Moreover, an abnormal Wnt signaling in adults has a role in the etiology of diseases such as cancer and bone diseases [69–72].

#### Structure

The Wnt family of ligands includes 19 types of secreted glygoproteins that are necessary for cellular transduction. Wnt ligands undergo post-translational modifications in the endoplasmic reticulum by glycosylation and palmitoylation prior to their release into the extracellular milieu where they activate signaling in further cells [69, 73, 74]. The extracellular movement of Wnts by simple diffusion is difficult because of their lipid modification. Hence, they need lipoprotein particles (LPP) acting as vehicle or they can interact with heparan sulfate proteoglycans (HSPGs) to facilitate their transport [74]. In the extra-cellular matrix, Wnt proteins stay inactivated through a diverse number of secreted proteins that bind to them and prevent their interaction with their receptors [69].

### Activation of Wnt pathway

#### Mode of activation

Wnt proteins reach their target cells where they bind to their receptors: Frizzled (Fz) receptors family. In humans, there are ten Fz protein members, each is a seven span transmembrane molecule with a long amino-terminal extension called CRD (cysteine-rich domain) and topological homology to G-protein coupled receptors (GPCR) on the cell surface. Wnt proteins bind directly to the extracellular N-terminal -CRD- of Fz receptors [75, 76]. The interaction between Wnt proteins and Fz proteins requires co-receptors such as the low-density-lipoprotein (LDL) related protein 5 or 6 (LRP5/6) mediating Wnt signaling and activating multiple downstream pathways [69, 74, 76].

The downstream components of Wnt signaling include Axin and Dishevelled molecules. The cytoplasmic tail of LRP co-receptor may interact directly with Axin [77]. Dishevelled (Dsh/Dvl) is the first cytoplasmic phosphoprotein that can directly interact with Fz while Dsh is involved in all three major branches of Wnt signaling leading to the formation of a complex including Fz recepror, LPR co-receptor, plus Axin and Dsh molecules. Then, Wnt signal branches into three major cascades according to the specific role of the cytoplasmic β-catenin protein, a hallmark of intracellular Wnt pathway activation. In the absence of Wnt signaling, β-catenin is phosphorylated by various proteins that form a “degradation complex,” allowing its ubiquitination and proteasomal degradation [69, 70, 75, 78].

#### Branches of Wnt signaling pathway

The first branch is the β-catenin dependent canonical Wnt pathway where the activation of Wnt signaling inhibits β-catenin phosphorylation so it remains in a stable form and not degraded. The elevated β-catenin levels in the cytoplasm lead to its nuclear translocation and complex formation with lymphoid enhancer factor/ T cell factor (LEF/TCF) transcription factors and activation of target genes. Canonical Wnt pathway has crucial roles in the regulation of diverse cell behaviors, including cell fate, proliferation, differentiation and polarity [72, 75]. Furthermore, it is the main component of Wnt signaling affecting bone cells [70].

The second branch is the non-canonical Wnt signaling pathway that is independent of β-catenin protein and does not lead to its cytoplasmic stabilization. It can be subdivided into two branches: Planar Cell Polarity pathway (PCP) pathway and Wnt/Ca^2+^ pathway having clear distinctions allowing it to be described as a separate branch. The canonical and non-canonical pathways often overlap to coordinate complex cellular responses [69, 74, 79].

The downstream events of the PCP pathway include binding of a Wnt ligand, such as Wnt5A: one of the most highly studied non-canonical members, to Fz receptors as well as a variety of co-receptors all of which contain a cysteine-rich binding domain. This interaction leads to a Dishevelled-mediated GTP-dependent activation of small GTPases which in turn activate c-Jun N-terminal kinase (JNK) regulateing cell polarity and gene transcription [80, 81].

Non canonical Wnt ligands can also trigger other β-catenin-independent effects through the regulation of intracellular Ca^2+^ concentration. The Ca^2+-^dependent pathway involves activation of protein kinase C (PKC) that increases intracellular Ca^2+^ concentration, triggering the activation of calcium/calmodulin-dependent protein kinase II (CaMKII) and nuclear factor of activated T cells (NFAT) pathway and transcriptional regulation. Ca^2+^/Wnt signaling has been mainly explored in the context of embryonic development, and its clinical relevance in adults is less clear [80, 82].

### Wnt signaling and osseointegration

#### Role of Wnt pathway in early and late osseointegratiom events

Wnt is one of the signaling pathways associated with oseteogenesis during differenet phases of osseointegration. It regulates osteoblast and mesenchymal stem-cell differentiation, hence its role in bone development [24, 40, 44]. Osseointegration starts with “contact osteogenesis” which is a direct contact between the roughened implant surface and newly formed woven bone, the first bone formed after bone injury. Then the presence of Titanium immune-modulates the following bone healing events [22, 44]. Woven bone is removed by osteoclasts and replaced by the load-oriented lamellar bone in the remodeling phase that can last for years after implant placement [24, 46]. New bone formation begins with osteoblasts secreting collagen matrix that is then mineralized by hydroxyapatite [46]. Since the canonical pathway is the predominant component of Wnt signaling affecting bone cells, many canonical Wnt ligands have essential role in bone homeostasis in general and help in increasing bone mass. For example, Wnt10b is an important ligand expressed by the bone marrow T lymphocytes. It is a positive modulator of bone formation, enhancing osteoblast differentiation and maintaining mesenchymal and/or osteoblast progenitors in adult bone [70, 83].

Bone remodeling is a complex process having osteoclasts and osteoblasts as key cellular players. Both act interdependently to achieve physiologic bone balance. Osteoclasts remove primary bone implant contacts creating space for new bone formation. They resorb bone without damaging the surrounding environment in the so called bone remodeling compartment. Then, the remaining surfaces left by osteoclasts become colonized by osteoprogenitor cells [40, 46]. Canonical Wnt signaling enhances mesenchymal stem cell (MSC) differentiation into osteoblasts and it represses osteoclast differentiation indirectly through the increased secretion of osteoprotegerin. Wnt ligands also possibly affect osteoclasts directly through an autocrine loop with a significant increase of Wnt10b ligand in mature osteoblasts [70, 84]. Osteoclasts may have a dual role in bone remodeling: they are responsible for bone resorption and they also recruit osteoprogenitors to the site of bone remodeling through sphingosine 1-phosphate (S1P) and bone morphogenic protein 6 (BMP6). Hence, they stimulate bone formation through increased activation of Wnt and BMP pathways [40, 84].

Osteocytes are mature osteoblasts that maintain bone homeostasis and regulate the action of osteoblasts and osteoclast during bone remodeling [70]. They regulate bone resorption and formation. They are the major source of RANKL in bone required for osteoclast differentiation and function and they function through Wnt signaling pathways regulating osteoblast proliferation, differentiation, and survival [40]. During bone remodeling, the newly formed lamellar bone has to be built in a load oriented fashion. Mechanotransduction is a major role of osteocytes that translate mechanical stimuli into cytokine signals to control osteoblast action. So, after implants functional loading, bone remodeling continues for a long time through the molecular signals from osteocytes [40, 46].

Functional mechanical loading is sensed by osteocytes whose mechanoreceptors regulate intercellular communication through gap junctions. Wnt signaling plays an essential role in osteocytes mechanosensing. Osteocytes are the main source of sclerostin, the soluble inhibitor of canonical Wnt signaling. Sclerostin expression is regulated by mechanobiology that is closely connected to the parathyroid hormone (PTH) signal transduction system. The absence of mechanical loading generates a low-strain signal and the bone becomes in a disuse state with subsequent bone loss [40, 46, 70].

#### Wnt pathway and different biological responses of bone types

A successful osseointegration is correlated with bone type. Type I and type III bone have different healing potentials in equivalent osteotomy sites. This was evident at day seven after implant insertion in a murine model where type III bone osteotomies were filled with mineralizing collagen matrix with a faster rate of new bone formation. This can be attributed to the positive correlation between bone cells endogenous Wnt responsiveness and the osteogenic properties of the bone type. Type III bone has significantly more Wnt stem/osteoprogenitor cells with a higher expression of Wnt target genes, than type I bone [85].

Implant failure with fibrous encapsulation and loss of osseointegration represents clinicians’ nightmare. Adequate implant insertion torque ensures primary stability that has been a predictor for successful osseointegration [40]. However, this was not always a straight forward causal relationship. Inappropriate loading and/or peri-implantitis may result in implant failure. Creating an environment with elevated Wnt signal around the implant was suggested to prevent such implant failure. Wnt reporter homozygous (*Axin2*^*LacZ/LacZ*^) mice were used. They represent a strong model of amplified endogenous Wnt signaling since they lack both copies of Axin2, the negative feedback regulator. Implants were placed in gap-type interfaces. Wild-type and *Axin2*^*LacZ/+*^ mice showed evidence of implant fibrous encapsulation while *Axin2*^*LacZ/LacZ*^ mice showed evidence of active bone mineralization around failing implants, suggesting that end-stage implant failure state may be reversible. Using a pro-osteogenic stimulus may improve the chances of implant osseointegration especially in those cases where anatomy, bone type or previous disease has compromised the implant bed [85, 86].

#### Wnt-based protein and gene therapy

The importance of Wnt–β-catenin pathway in adult bone homeostasis suggested its importance as an osteoanabolic drug target. Targeting Wnt antagonists such as sclerostin and dickkopf 1 (DKK1) was achieved in animal models using therapeutic proteins to treat bone diseases [70]. Similarly, the role of Wnt signaling in osseointegration opened a new therapeutic potential to rescue failed oral implants. Purified Wnt3A protein sustained in vivo activity and thus could be tested in a clinically relevant model of implant osseointegration. It was found that adding exogenous liposomal Wnt3A (L-Wnt3A) protein induced strong Wnt response in peri-implant cells transforming implants with fibrous encapsulation into osseointegrated ones in a mouse model [85, 87, 88].

These results were confirmed in another study using an ovariectomized (OVX) rat model. Peri-implant injections of L-Wn3A or a liposomal formulation of phosphate buffered saline (L-PBS) as a control were performed at the time of implant placement. Finite element model coupled with molecular and cellular analyses revealed that the expression of Axin2 -a direct Wnt target gene- was upregulated. At day 14, all peri-implant cells showed evidence of osteogenic differentiation and mineralization. At the same time point, lateral stability testing confirmed that the L- Wnt3A treated cases have stiffer interface than the control group [89].

Osteoporosis is a disease affecting hundreds of millions of people worldwide, particularly postmenopausal women and older men. It is characterized by decreased bone density and strength so the skeleton cannot adequately perform its support function. Osteoporosis-pseudoglioma syndrome is a specific type of osteoporosis linked to loss-of-function mutation in *LRP5* encoding for the Wnt co-receptor LRP5 [70, 90]. The Wnt pathway is not only involved in bone homeostasis, it has also an essential role in glucose homeostasis and lipid metabolism. Mutations in *LRP5* were linked also to the onset of diabetes and obesity [91]. Diabetic patients are more prone to osteoporosis since they suffer from a deficient metabolism of skeletal tissue because of suppressed osteoblastic function with lower potential of bone formation. Furthermore, uncontrolled diabetic patients have a higher risk of implant failures [40].

Peroxisome proliferator-activated receptors (PPARs) are ligand-activated transcription factors playing a major regulatory role in energy homeostasis and metabolic function. Activation of the subtype PPAR-γ causes insulin sensitization and enhances glucose metabolism. PPARs represent interesting therapeutic targets for many diseases including diabetes [92, 93]. In an attempt to overcome the detrimental effects of diabetes and the associated osteoporosis on the osseointegration of dental implants, PPARγ gene delivery was tried in a diabetes mellitus induced rat model having a lower PPARγ expression. Chitosan gold nanoparticles conjugated with PPARγ cDNA were introduced on the surfaces of Ti mini-implant placed in the rat’s mandible. PPARγ gene delivery activated mitochondrial biogenesis and cell viability through the p-AMK and Wnt/β-catenin signaling with enhanced osseointegration and new bone formation [94].

Animal models showed that the expression of Wnt pathway genes was significantly downregulated in ovariectomized (OVX) rats. Human Wnt10b (hWnt10b) expressed from an adenovirus vector was locally delivered to the femoral defect site in OVX rats prior to implant placement. OVX rats treated with Ad-hWnt10b showed markedly increased ALP, Runx-2, and osteocalcin expression and decreased cathepsin K expression. Meanwhile, Ad-hWnt10b-BMSCs showed significantly increased osteogenesis and decreased adipogenesis. So, hWnt10b may accelerate osseointegration around implants and enhance bone regeneration and implant stabilization under OVX conditions [95]. The same concept may be applied to dental implants. More studies are needed.

#### Canonical Wnt signaling, macrophages and implant’s surface characteristics

Macrophages are key cellular players in the inflammatory, proliferative and remodeling phases after implant placement [46]. A proper balance between the pro-inflammatory M1 and pro-regenerative anti- inflammatory M2 macrophages phenotypes is essential for a proper osseointegration. Macrophages differentiate from circulating monocytes into M1 or M2 phenotypes depending on adjacent biomaterial surface characteristics. Studies have demonstrated that rough and hydrophilic titanium surfaces direct the differentiation of macrophages towards an anti-inflammatory M2-like phenotype as compared to smooth surfaces. M2 macrophages can produce Wnt ligands activating the osteogenic Wnt canonical pathway, hence their essential role in osteoimmunomodulation [22, 96].

In fact, the relation between Wnt signaling and macrophages is more complicated and can be described as an autocrine loop. Wnt signaling regulates macrophage response to biomaterials and in the same time macrophages are an important source of Wnt ligands during inflammation and healing. Wnt signaling plays a pivotal role in both classical and surface-mediated macrophage activation and macrophages significantly contribute to Wnt signaling in the peri- implant microenvironment. Biomaterial surface properties modulate Wnt signaling gene expression in macrophages. Rough implant surfaces resulted in increasing Wnt ligand production in macrophages. Wnt ligands can induce the production of pro- and anti-inflammatory cytokines characteristic of polarized macrophages [97].

A recent study investigated if Wnt/β-catenin signaling can direct macrophages activation and polarization towards a pro-inflammatory state. Smooth and rough-hydrophilic Ti surfaces were used based on the fact that both surface modifications generate opposite macrophage phenotypes. The results showed that Wnt/β-catenin signaling activation enhances a pro-inflammatory macrophage polarization and microenvironment. Canonical Wnt signaling increases the availability of important interleukins and TLRs that play an important role in macrophages polarization. Hence, macrophages respond to inflammatory signals activating them into a pro-inflammatory state creating an inflammatory microenvironment at the implant site [98].

#### Effect of implant surface modifications on Wnt signaling

Implant’s surface characteristics have a great influence on the adjacent cells’ adherence, morphogenesis, differentiation and gene expression. The cell/material interaction differs between polished smooth surfaces and rough SLA hydrophobic surface or chemically modified SLA surface having high wettability/hydrophilicity (SLActive) [43]. The molecular mechanisms of osseointegration were studied on animal models. Results showed that gene expression of all biologically relevant processes varied in relation to smooth and rough implant surfaces. Genes related to skeletogenesis, mesenchymal cell differentiation, angiogenesis and neurogenesis were studied. The main signaling pathway that was differentially regulated between the two surfaces was the Wnt signaling pathway [44, 99].

The cellular and molecular responses of human bone marrow-derived mesenchymal stem cells (hMSCs) also vary in relation to implant’s surface characteristics. Rough SLA and SLActive surfaces showed earlier calcified matrix deposition than smooth surfaces, with increased gene expression of Wnt5A promoting osteogenesis in hMSCs [100]. Wnt3A ligand increases hMSC proliferation and promotes stem cell renewal by activation of the Wnt/β-catenin pathway. This applies to smooth surface implants only since Wnt3a has no effect on rough implant surfaces. Wnt5A increases commitment of hMSCs to an osteogenic linage on microstructured rough titanium implants surfaces. It induces osteoblast differentiation via the Wnt calcium-dependent pathway [101].

**Fig. 2:**
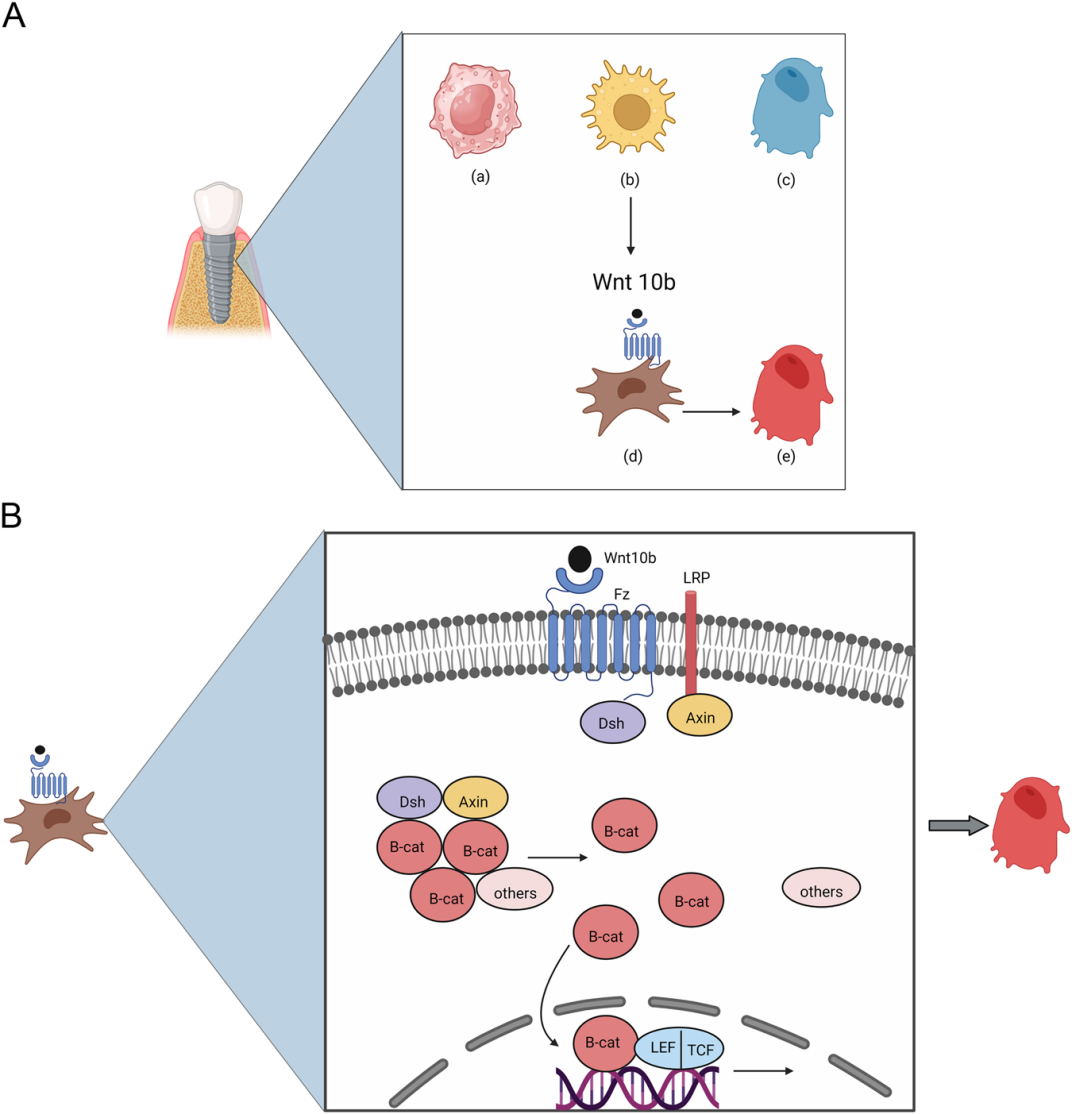
Canonical Wnt pathway and osteoblasts differentiation around dental implants. **A**, **B** Summarizes the role of Wnt/β-catenin pathway in osteoblasts differentiation. **A** Diagrammatic representation of a dental implant surrounded by alveolar bone during late osteogenic osseointegration events. At the implant bone interface, (a) M2 macrophages, (b) activated T cells and (c) mature osteoblasts secrete Wnt10b ligand responsible for the commitment of (d) mesenchymal stem cells to osteogenic lineage. Wnt10b ligand attaches to Frizzled (Fz) receptor at the cell membrane, in the presence of LRP co-receptor, activating the canonical Wnt/β-catenin pathway inside the cell leading to its differentiation and maturation into (e) osteoblast. Created with BioRender.com. **B** Wnt10b ligand activation of Fz receptor leads to B-catenin complex degradation. Dishevelled (Dsh) is the first cytoplasmic molecule that attaches to the tail of Fz receptor. The cytoplasmic tail of LRP co-receptor interacts directly with Axin molecule. Canonical Wnt signaling inhibits β-catenin phosphorylation so it remains in a stable form and not degraded. The elevated β-catenin levels in the cytoplasm lead to its nuclear translocation and complex formation with lymphoid enhancer factor/ T cell factor (LEF/TCF) transcription factors and activation of target genes. Created with BioRender.com.

Figure 2A and B summarizes the role of Wnt/β-catenin pathway in osteoblasts differentiation.

## Cross-talk between NF-κB and Wnt pathways

The functions of an individual signaling pathway are extended through its crosstalk with another one resulting in a more complex regulatory network. Recent studies found that the NF-κB and the Wnt/β-catenin pathways cross-regulate their activities and functions positively and negatively. The underlying molecular mechanisms depend on the cell type and environmental stimuli [102]. The presence of implants in direct vicinity to relevant cells may play a role in this cross-talk.

Wnt signaling modulates macrophage polarization into M1 or M2 phenotypes and it is regulated by the implant’s biomaterial surface properties. Therefore, canonical Wnt signaling has pro and anti-inflammatory functions [97, 98]. NF-κB is a key transcription factor of the pro-inflammatory M1 macrophages, while M2 macrophages are anti- inflammatory pro-regenerative cells that produce Wnt ostegenic ligands [22, 30, 52]. This can be the way both pathways mutually affect each other through variations in the implant’s surface characteristics. Studies are needed to evaluate their cross-talk in relation to the osseointegration of dental implants.

## Future prospectives: personalized dentistry and implantogenomics

Advances in molecular biology and “omics” technologies paved the way to uncover molecules and signaling pathways involved in complex biological processes. Comprehensive gene expression analyses and proteomic studies were able to identify early and late molecular events of osseointegration in relation to different implant’s surface characteristics. Omics studies helped physicians in targeting relevant proteins by new therapies and targeting defective genes through gene therapy in a personalized medicine approach. The same applies in the dental field where future application of omics technologies will be in personalized dentistry and implantology [103].

Personalized/precision medicine is an evolving healthcare strategy aiming to tailor a specific medical treatment following the patient’s individual genetic, anatomical and physiological characteristics. The same can be applied to the dental field where personalized dentistry gained recent attention, especially in dental implants therapy where the biological concepts -like the signaling pathways- play a pivotal role in the success or failure of implant’s therapy [104].

Implantogenomics is a new term that was proposed along with the concept of personalized dental implant therapy. Similar to the pharmacogenomics concept in medicine, implantogenomics should fulfill three major components before being applied in clinical practice to achieve a personalized implant treatment. These are:

- First; patients’ stratification according to genetic variations most relevant to osseointegration. Variations in the genes encoding the main transcription factors of NF-κB and Wnt pathways as well as variations in their target genes should be included.
- Then identifying biomarkers predicting the prognosis of implant healing in each patient’s subpopulation.
- Finally, the third component is the idea of designing an implant that suits each subpopulation following their genetic constitution [104, 105].

This concept offers challenging opportunities for multi-disciplinary research combining multi-omics fields with advanced material science and biotechnology using recent bioinformatics and artificial intelligence tools. A long road of research is needed to reach this optimistic goal and develop the new field of implantogenomics or implantomics [104]. Recent advanced omics technologies enabled better understanding of cell-to-cell communication and gene in the implant microenvironment [106]. Further studies on osseointegration on a molecular level, using the available knowledge of relevant signaling pathways can pave the way to develop an applicable personalized dental implant therapy.

## Conclusion

Recent bioinformatics technologies could reveal the secrets of complex cellular transduction pathways involved in all aspects of biological processes; osseointegration is one of them. NF-κB pathway regulates early immuno-inflammatory osseointegration events whereas the later osteogenesis-related mechanisms are regulated by Wnt signaling. Both cross-talking pathways are affected by the implant’s material and surface chracteristics. Both pathways also greatly influence the key cellular players of osseointegration. Wnt-based protein and gene therapy studies offer a new hope to treat failed implants and enhance osseointegration in difficult cases having poor prognosis. This is consistent with advances in Omics technologies that can change the paradigm of dental implant therapy.

## Data Availability

Data availability statement is not applicable to this study since as it is a narrative review.
